# Bipartite graph search optimization for type II diabetes mellitus Jamu formulation using branch and bound algorithm

**DOI:** 10.3389/fphar.2022.978741

**Published:** 2022-08-11

**Authors:** Wisnu Ananta Kusuma, Zulfahmi Ibnu Habibi, Muhammad Fahmi Amir, Aulia Fadli, Husnul Khotimah, Vektor Dewanto, Rudi Heryanto

**Affiliations:** ^1^ Department of Computer Science, Faculty of Mathematics and Natural Sciences, IPB University, Bogor, Indonesia; ^2^ Tropical Biopharmaca Research Center, IPB University, Bogor, Indonesia; ^3^ Department of Chemistry, Faculty of Mathematics and Natural Sciences, IPB University, Bogor, Indonesia

**Keywords:** branch and bound, diabetes mellitus, drug–target interaction, graph traversing, jamu

## Abstract

Jamu is an Indonesian traditional herbal medicine that has been practiced for generations. Jamu is made from various medicinal plants. Each plant has several compounds directly related to the target protein that are directly associated with a disease. A pharmacological graph can form relationships between plants, compounds, and target proteins. Research related to the prediction of Jamu formulas for some diseases has been carried out, but there are problems in finding combinations or compositions of Jamu formulas because of the increase in search space size. Some studies adopted the drug–target interaction (DTI) implemented using machine learning or deep learning to predict the DTI for discovering the Jamu formula. However, this approach raises important issues, such as imbalanced and high-dimensional dataset, overfitting, and the need for more procedures to trace compounds to their plants. This study proposes an alternative approach by implementing bipartite graph search optimization using the branch and bound algorithm to discover the combination or composition of Jamu formulas by optimizing the search on a plant–protein bipartite graph. The branch and bound technique is implemented using the search strategy of breadth first search (BrFS), Depth First Search, and Best First Search. To show the performance of the proposed method, we compared our method with a complete search algorithm, searching all nodes in the tree without pruning. In this study, we specialize in applying the proposed method to search for the Jamu formula for type II diabetes mellitus (T2DM). The result shows that the bipartite graph search with the branch and bound algorithm reduces computation time up to 40 times faster than the complete search strategy to search for a composition of plants. The binary branching strategy is the best choice, whereas the BrFS strategy is the best option in this research. In addition, the the proposed method can suggest the composition of one to four plants for the T2DM Jamu formula. For a combination of four plants, we obtain *Angelica Sinensis*, *Citrus aurantium*, *Glycyrrhiza uralensis*, and *Mangifera indica*. This approach is expected to be an alternative way to discover the Jamu formula more accurately.

## Introduction

Jamu, known as Indonesian herbal medicine, is local wisdom that must be preserved because it has been practiced for generations (Elfahmi et al., 2014). The 2010 Basic Health Research results show that more than 50% of Indonesians use herbal medicine (Purwaningsih, 2013). Jamu is made from various plants that are considered to have healing properties based on practical experience. Zuhud et al. (2001) identified approximately 1,845 forest plant species in Indonesia that have the potential as medicinal plants. National Agency of Drug and Food Control in Indonesia noted that approximately 283 plant species were officially registered and used for treatment. Thus, Jamu has the potential to be developed. Because of the vast biodiversity of Indonesia’s indigenous medicinal plants, herbal medicine has the potential for economic development (Elfahmi et al., 2014). However, this herbal medicine has not been widely used because the discovery of herbal formulas has not been supported by its scientific basis (Noor et al., 2022).

Various efforts to make herbal medicine have a computational-based scientific basis have been carried out. Research on herbal medicine by Afendi et al. (2010) put forward the hypothesis that at least one Jamu formula has a composition of four herbal plants. One main plant directly affects disease, and the other three are supporting plants that have analgesic, antimicrobial, and anti-inflammatory properties. The 3138 herbal formulas taken from 465 plants were classified into nine properties (Afendi et al., 2010). Afendi et al. (2013) continued their research by looking for the relationship between plant composition and herbal medicine efficacy using a statistical approach to classify nine properties of the 3138 Jamu formulas derived from these 465 plants. Classification based on partial least squares discriminant analysis produced an accuracy of 71.6%. Fitriawan et al. (2013) conducted a similar study with a machine learning approach using the support vector machine (SVM) method, resulting in an accuracy of 71%. Puspita et al. (2016) conducted another study that reported the study of feature selection using clustering techniques to reduce the number of irrelevant features before training using SVM.

Prediction of herbal composition based on plant composition still does not obtain high accuracy. In addition, formula predictions based on plant composition cannot describe the interaction mechanism between compounds contained in plants and target proteins that represent certain diseases. The network pharmacology approach, first presented by Hopkins (2008), provides an opportunity to investigate the molecular complexity of herbal formulas and the correlation between herbal formulas and disease complexes (Wu et al., 2013; Du et al., 2014). It has been shown to work in various herbal compositions used in traditional medicine (Emig et al., 2013; Lotfi Shahreza et al., 2018). Furthermore, in this big data era, we can repurpose traditional medicines by analyzing the combinatorial properties of herbal formulas and their mechanism of action (Newman et al., 2008; Huffman and Shenvi, 2019). With the rapid advances in bioinformatics and systems biology, network-based drug discovery is seen as a promising approach to more cost-effective drug discovery (Keith et al., 2005; Jia et al., 2009; Schadt et al., 2009; Zhang et al., 2019; Chaudhari et al., 2020; Noor et al., 2022).

One of the representations of network-based drug discovery is drug–target interaction (Li et al., 2009). Many studies predicted interactions between compounds and target proteins, such as using machine learning techniques, classification algorithms, learning to rank algorithms, and deep learning algorithms (Xu et al., 2021). Yamanishi et al. laid the basis for drug–target interaction (DTI) prediction research. Their systematic study employed a bipartite local model based on an SVM Yamanishi et al. (2008). Yamanishi et al. (2010) used a distance learning algorithm as a classifier. Other studies have used a binary classification approach with machine learning techniques, such as SVM and random forest (RF), to predict drug or compound interactions with target proteins (Nasution et al., 2019; Shi et al., 2019; Erlina et al., 2020; Wijaya et al., 2021). In this binary classification approach, the features that represent DTI are obtained from the compound fingerprint and the descriptor of the protein. For example, in Erlina et al. (2020), the PubChem fingerprint was used for its compound consisting of 881 features and a dipeptide descriptor consisting of 400 features. The total number of features is 1281. Therefore, Erlina et al. (2020) reported that the binary classification model for this DTI faces high-dimensionality problems that affect the model’s accuracy.

Several studies used deep learning to predict this DTI (Fitriawan et al., 2016; Lee et al., 2019; Mei and Zhang, 2019; Sulistiawan et al., 2020; Sajadi et al., 2021). Lee et al. (2019) proposed a deep learning-based prediction model capturing local residue patterns of proteins participating in DTIs. This was motivated by reports about conventional learning-based prediction models being not informative in predicting accurate DTIs. Sajadi et al. (2021) proposed a method based on deep unsupervised learning for drug–target interaction prediction called AutoDTI++ to solve the sparsity problem of the interaction matrix. Sulistiawan et al. (2020) used stacked autoencoder (SAE) as pretraining for initializing weights on the deep neural network (DNN) to prevent learning from stopping too quickly. SAE for DNN pretraining can prevent the layer outputs from vanishing during the training process (Boulila et al., 2021) and help to achieve better generalization in prediction results (Bahi and Batouche, 2018). However, there is a drawback to using binary classification for predicting DTI. It simplifies the DTI issue by modeling high-dimensional compound–protein and their complex associations into a binary classification model without considering the relationship between compounds or proteins (Mei and Zhang, 2019).

Thus, Pliakos et al. (2019) and Fadli et al. (2021) used multilabel classification based on deep learning to generate a prediction model for DTI. Multilabel classification can be used to solve binary classification problems. In multilabel classification, the training process produces a model that maps input vectors to one or more classes. The prediction of the target is only determined based on the pattern of the existing compound structure. Utilizing proteins as class labels can reduce the input dimensions because it does not require feature extraction of the protein. In addition, from a machine learning perspective, apart from being able to predict several interactions at once, the multilabel classification model can identify possible correlations between class labels (proteins) to increase the performance of DTI predictions (Pliakos et al., 2019).

The approach to machine learning for DTI, whether using conventional machine learning methods, ensemble methods, or deep learning, raises an important issue. Its application to predict the formula of herbal compounds for certain diseases often leads to different results. Erlina et al. (2020) reported the results of different herbal compounds using multilayer perceptron, SVM, and RF. To conclude which compounds and target proteins have the most potential, Erlina et al. (2020) analyzed the overlapping predictions of herbal compounds across all methods. Likewise, Fadli et al. (2021) have built four different models based on compound features. The four models produce several different predictive compounds, so it is necessary to perform an overlapping analysis of the predicted results. Another limitation of the machine learning approach in predicting the Jamu formula is that we cannot immediately know what plants contain these candidate compounds. To get plant information, we have to do a literature study or look for it in databases, such as KNApSAcK (Afendi et al., 2012) and IJAH Analytics (http://ijah.apps.cs.ipb.ac.id).

This research proposes a new approach using the graph traversing technique to overcome the limitation of machine learning approaches. In this study, a tree representing DTI was built, and the unknown interactions were determined based on similarity measurements among compounds and proteins that meet a specific threshold value. Furthermore, the bipartite network representing DTI was expanded into a bipartite plant–protein network. Moreover, we applied a graph traversing algorithm with the branch and bound technique to perform tree searches to find medicinal plants for certain diseases. Morrison et al. (2016) stated that the branch and bound algorithm has been used successfully to find exact solutions for a wide area of optimization problems. Zhang et al. (2012) used branch and bound with a bipartite graph to solve the single vehicle routing problem with a toll-by-weight scheme. The results showed that branch and bound outperforms the best-known exact algorithms at that time for the unweighted minimum latency problem and was able to find the optimal solution. Wang et al. (2019) used the branch and bound technique to traverse the bipartite graph of resource allocation problems in radio broadcast scheduling, and the results showed that the algorithm greatly reduces the searching space and execution time. In bioinformatics, Sridhar et al. (2008) demonstrated branch and bound usage to explore the metabolic networks and find the target for known successful drugs. The algorithm can accurately identify the target enzymes that interacted with the drugs and reduce the total search time compared with the exhaustive search. Zhou et al. (2016) used a modified branch and bound algorithm to find the global minimum energy conformation in structure-based computational protein design. The algorithm is able to exploit the structure of residue–residue interaction graph to significantly accelerate the process. Thus, we proposed to use the branch and bound technique because of its ability to find the solution optimally while being able to reduce search time and space. To show the performance of the proposed method, we compared our method with the complete search algorithm, which searched all nodes in the tree without pruning. In this study, we specifically apply the proposed method to search for the Jamu formula for type II diabetes mellitus (T2DM) disease. T2DM is a disease characterized by carbohydrate, fat, and protein metabolism disorders and a lack of work and insulin secretion (Fatimah, 2015). We hope that the proposed method will become an alternative method for predicting interactions in drug–target and for searching the Jamu formula for T2DM.

## Materials and methods

### Data acquisition

Data acquisition was done using web crawling techniques on several databases and related research results. These data were used to build three pharmacological networks as follows.

1) Network A represents a plant–compound–protein network. The plant data are taken from the KNApSAcK database (Afendi et al., 2012). The compound data are taken from PubChem (Kim et al., 2019) and KNApSAcK database. The protein target data are taken from PubChem BioAssay (Wang et al., 2017).
2) Network B is an extension of network A by adding 10 compounds obtained from searching over compounds in the ChemMine-Tools database (Backman et al., 2011). These 10 compounds have similarity scores of at least 0.9 to each compound in network A.
3) Network C represents the relationship between the T2DM target proteins in Uniprot and the compounds in PubChem BioAssay.

**FIGURE 1:**
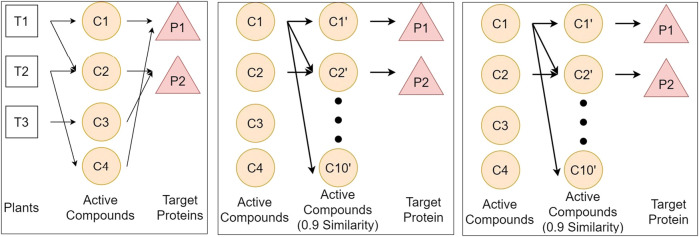
Illustration of three networks of plants, compounds, and proteins, respectively represented by T, C, and P. We define three networks from different databases to get the relationship between plants and protein. Network A connects plants from KNApSAcK, compounds from KNApSAcK and PubChem, and proteins from PubChem BioAssay. Network B connects compounds in network A, compounds in network B taken from ChemmineTools, and the target protein is the same as that in Network A. Network C connects proteins from Usman et al. (2020), proteins from Uniprot, and compounds from PubChem BioAssay.

Figure 1 illustrates the three above-mentioned networks. The details of data and source databases for each network are provided in Table 1.

**TABLE 1:** Results of data acquisition from the various databases.

Network	Data	Data resources	Results
A	Plants	KNApSAcK	- 711 plants
	Compound	KNApSAcK	- obtained 4926 compounds from 711 plants with 7725 interactions of plant–compound
			- only 581 plants have at least one compound
	Compound	PubChem	- only 2780 of 4926 compounds have CID and are categorized as compound
			- only 541 plants have at least one compound
	Target protein	PubChem BioAssay	- obtained 2308 target proteins with 131.798 interactions of compound–protein
			- only 1063 compounds have at least one target protein
B	Compound	ChemmineTools	- obtained 9647 compounds from the expansion of network A
	Target protein	PubChem BioAssay	- obtained 2465 target protein from 9647 compound
C	Target protein	Usman et al. (2020)	- 21 target proteins associated with T2DM
			- The score of betweenness centrality (BC) and closeness centrality (CC)
	Target protein	UniProt	- MGI to GI id conversion for each target protein
	Compound	Pubchem BioAssay	- obtained 803 compounds have interaction with 14 target proteins of T2DM

Each compound has a CID and a CAS ID. The CAS ID is used to find a CID that corresponds to the compound in the PubChem database.

### Data preprocess

The data was preprocessed on the target protein of T2DM because of Usman et al. (2020). There are 21 proteins, each has the betweenness centrality (BC) and closeness centrality (CC) values. The two values are averaged and then normalized to the range of 0–1. This value becomes the weight of a protein. Table 2 shows the normalization results. Genes in Table 2 are attributes that indicate the gene name of the T2DM protein. BC and CC are the BC and CC, respectively, whereas AVG is the average value of BC and CC. The normalization results are shown in the NORM column. From the data acquisition results, only 14 T2DM proteins could be targeted by at least one compound. Therefore, we carried out analysis and experiment with those 14 T2DM target proteins.

**TABLE 2:** Protein weight normalization results.

Gene	BC	CC	AVG	NORM
INS	0.3211	0.6250	0.4731	1.000
AKT1	0.2435	0.5128	0.3782	0.799
TCF7L2	0.2003	0.5714	0.3859	0.816
KCNJ11	0.1342	0.5000	0.3171	0.670
UBC	0.1097	0.4878	0.2987	0.632
PPARG	0.0952	0.5128	0.3040	0.643
GCGR	0.0780	0.4762	0.2771	0.586
INSR	0.0775	0.5000	0.2888	0.610
IAPP	0.0526	0.4348	0.2437	0.515
SOCS3	0.0518	0.4348	0.2433	0.514
EP300	0.0443	0.4167	0.2305	0.487
PPARA	0.0311	0.4082	0.2197	0.464
WFS1	0.0186	0.4444	0.2315	0.489
APOE	0.0163	0.3846	0.2004	0.424
FOXO1	0.0096	0.3704	0.1900	0.402
STAT3	0.0066	0.3509	0.1787	0.378
PTH	0.0044	0.3509	0.1776	0.375
CTLA4	0.0000	0.3448	0.1724	0.364
MTNR1B	0.0000	0.3922	0.1961	0.414
PRKACA	0.0000	0.3390	0.1695	0.358
SOD3	0.0000	0.3448	0.1724	0.364

### Measurement of compound similarity

Measurement of the similarity of two compounds was carried out using the Tanimoto coefficient. The Tanimoto coefficient is used to measure the degree of similarity with the formula. The more similar the two compounds are, the higher their Tanimoto coefficient. To be specific, the Tanimoto coefficient approaches 1 when two compounds have more similarities. By contrast, the Tanimoto coefficient approaches 0 when two compounds have more dissimilarities. For this reason, the compound structure is encoded into binary bits representing the compound’s molecular structure. We utilized the Klekota–Roth fingerprint, which has 4860 binary features. For each compound, the fingerprint algorithm encodes 1 in a bit if there is a corresponding molecular structure, and 0, otherwise. Eq. 1 shows the Tanimoto coefficient formula.

$$\mathrm{coef}=\frac{c}{\left(a+b-c\right)}$$ (1)

where *a* denotes the number of bits 1 in the first compound.*b* denotes the number of bits 1 in the second compound.*c* denotes the number of 1 bit in both compounds.

In this study, we used the fingerprint algorithm to calculate the similarity score between the compounds in networks A and C.

### Connecting networks A, B, and C

The three main networks from this research are networks A, B, and C, as shown in Figure 1. The vertex or node of the network is a component, which can be a plant, a compound, a protein, or a disease, whereas the edge represents the connection between components. We used an adjacency list data structure to store the networks. An adjacency list is a data structure that stores graphs like a neighbor list. By using this data structure, we can speed up the tracing process because enumerating a vertex’s neighbors can be done in O(k), where k is the number of neighbors of a vertex (Blandford et al., 2003).

#### Networks A and B connection

Networks A and B are connected because network B is an extension of network A. The expansion is through the similarity between compounds in network A (denoted as C_a_) and compounds in network B (denoted as C_b_).

#### Networks A and C connection

Network A (denoted as N_a_) and C (denoted as N_c_) are connected through two pathways, namely, compound similarities and protein similarities. The compound similarity was formed by calculating the similarity of each compound in network C (denoted as C_c_) with the compound in network A (C_a_) using the Klekota–Roth fingerprinting and Tanimoto coefficient. For each compound in network C (C_c_), we record all compounds with the highest similarity score and create a new edge between C_a_ and C_c_. The pseudocode is provided in Supplementary Figure S1.

The protein similarity pathway was formed by looking at the proteins in networks A and C that are the same. For each exact protein pair, a new edge is created between the two proteins. The pseudocode is provided in Supplementary Figure S2.

#### Networks B and C connection

Networks B and C are linked by protein similarity. A new edge is created between every protein in network B that is the same as the protein in network C. The pseudocode is provided in Supplementary Figure S3.

### Graph traversing for constructing a weighted bipartite network plant–protein

Graph traversing from T2DM proteins to plants aims to determine which plant can target T2DM proteins. For this process to be efficient, it is necessary to trace the T2DM protein to the compound in network A. Then, we stored the interaction information between proteins and compounds in network A. Next, for each compound, we traced it back to the plant containing the compound in network A. Any components (plants, compounds, or proteins) that cannot be traced from the T2DM protein was removed from the network to form a simpler network so that the search process became more efficient.

Next, we conducted graph traversing to construct a bipartite network of plant–compound–protein as follows:

1) Traverse from network C to network B

A complete search was started by searching from network C to network B. At first, the compound in network C was removed. Then, a search was carried out from the T2DM proteins (denoted as P_c_) to each compound in network B (C_b_). First, look for P_c_ and P_b_ that are the same, where P_b_ denotes proteins in network B. Next, store information on which target protein (P_t_) traced each compound C_b_. Again, a search was carried out from the T2DM proteins (P_c_) to each compound C_b_ and recorded any target protein (P_t_) connected with compound C_a_ whose similarity weight to compound B (C_b_) is at least 0.9. The pseudocode is provided in Supplementary Figure S4.

2) Traverse from network C to network A.

First, we removed the compounds in network C. A search was carried out from the T2DM proteins P_c_ to the compound C_a_ in network A. Then, we stored any target protein that is connected to all compounds C_a_. If there was a stored target protein (P_t_) connected to the compound C_a_, the previously recorded weight is updated to 1. Similar to the previous step, we traced the T2DM proteins (P_c_) but retained all compounds in network C. For each compound C_a_, record the T2DM protein (P_c_) as protein target P_t_ that traced it (if the protein has not been recorded previously), and update the edge weights if the similarity score of C_a_ and C_c_ traversed is greater than the previous edge weight. Last, backtracking was carried out to all compounds up to plants in network A. For each plant, if the weight of the target protein is greater than the weight of the previous target protein (if it has been recorded), then update the weight and record all the target proteins that had interaction with compounds traceable from the plant. The pseudocode is provided in Supplementary Figure S5.

Graph traversing over networks A, B, and C finally produces a relationship between plants and proteins. This relationship is represented as a weighted bipartite graph between plants and proteins. From the graph search results, some compounds and plants cannot target any T2DM protein. These components were eliminated from the network, leaving 1467 compounds and 460 plants in network A. Each compound and plant pair in network A has information in the form of any T2DM protein that can be traced, along with edge weights found during tracing the protein.

Examples of the search results and the stored information:

′73399′: ({′60391226′: [′Akt1′, 0.7993787198, 0.9]}, set([′60391226′])).

Information:

1) 73399 is CID a compound.
2) 60391226 is a GI of T2DM protein that can be traced from this compound.
3) Akt1 is the symbol gene for the T2DM protein.
4) 0.7993787198 is the weight of the T2DM proteins.
5) 0.9 is the edge weight that is passed when tracing the T2DM protein.
6) set([′60391226′]) is a set data structure to prevent double counting.

If the node is a plant, the information that changes is only the CID of the compound in the Latin name of the plant in question. For example, “*Schisandra chinensis Baill*.”: ({′60391226′: [′Akt1′, 0.7993787198, 0.9]}, set([′60391226′])). The difference with the previous result, namely, “*Schisandra chinensis Baill.*” is the plant’s Latin name.

### Composition of k plants as a candidate for herbal formula

Each plant has a relationship with one or more target proteins of TD2M. Each relationship has a different value. The greater the value of the relationship between a plant and protein indicates that the plant is associated with the target protein. In addition to the correlation value, each significant protein in T2DM has its weight. The known correlation value and protein weight will be used as a benchmark in calculating the herbal formula score using Eq. 2.

$$\mathrm{Formula}\_\mathrm{score}=\sum P_{i}W_{i}\;$$ (2)

where P_i_ denotes protein weight *i*th and W_i_ denotes edge weight of protein *i*th.

The higher the score of a formula, the better the formula will be in treating T2DM. If T plants and k unique plants are selected, there will be C(T, k) possible herbal formula candidates with k constituent plants. C(n, r) is a function that returns the value of the number of combinations of r objects from n objects. It shows that the memory and time complexity in finding the combination of k plants that make up the herbal formula is O(T^k^). However, memory usage optimization can be done by limiting the number of candidate herbal formulas. For example, if we only want an F for herbal formulas with the highest score, we can use a priority queue data structure to store the candidate herbal formulas. A priority queue is a data structure in the form of a (binary) heap. The (binary) heap itself is a complete binary tree. This data structure has characteristics: for each subtree with root X, the left and right child subtrees are smaller (or equal to) X. The complexity of inserting and popping data in the priority queue is O(log n), where n is the number of data stored in the priority queue, whereas the top process has a complexity of O(1).

The top is retrieving data with the maximum value in the priority queue, whereas the pop is removing data with the maximum value from the priority queue. This characteristic can be used to store the score of herbal formula candidates. Hence, the data stored in the priority queue is the herbal candidate with the highest score’ the score will be stored in the form of −score. This is because if the priority queue already accommodates F candidate herbal formulas, then the pop process will issue the herbal formula with the lowest score (−highest score).

With this technique and data structure, the memory complexity becomes O(F), where F is the number of candidates with the highest score. If this process is paralleled with t threads, then it takes t priority queues, each of which accommodates F herbal candidates. The t priority queues will then be combined into a priority queue. Memory complexity becomes O(t*F).

By contrast, the time complexity is still O(T^k^) because it must produce all combinations of k plants as candidate herbal formulas. However, optimization can be done by reducing T. Because the herbal formula to be sought has the highest score, plants that do not have the maximum edge weight for a T2DM protein can be eliminated. Then, search for plants that are not a subset (smaller) of T2DM protein than other plants, i.e., if we choose a plant X whose T2DM protein is a subset of another plant Y with more T2DM protein, then it is more optimal if we choose plant Y.

### Graph traversing using branch and bound technique

According to Morrison et al. (2016), branch and bound is a fundamental methodology and is widely used in solving exact solutions for NP-hard optimization problems. Branch and bound implicitly generate all possible solutions to the problem by storing partial solutions called subproblems in the tree structure. Unexplored nodes in the tree generate branches and partition the solution space into smaller regions that can be solved recursively (branching), and a pruning rule is used to reduce the search space size that proves to be nonoptimal (pruning). In the branch and bound algorithm, three components are not explicitly explained but can significantly influence the algorithm’s performance. These components are search strategy, branching strategy, and pruning rules.

### Searching strategy

**FIGURE 2:**
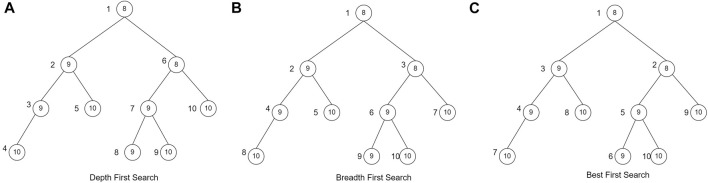
Searching strategy (Morrison et al., 2016)

In this study, the branch and bound technique will be implemented using the search strategy of breadth-first search (BrFS) (Bundy and Wallen, 1984), depth-first search (DFS) (Morrison et al., 2016), and best-first search (BFS) (Morrison et al., 2016). The difference between these three search strategies is the order in which the nodes are searched. BrFS performs a search by searching for the nearest neighbor, or in this case, the nearest neighbor is a child of that node. A search by visiting the nearest neighbor will make the search comprehensive. Next, the second strategy is DFS. A search on DFS will perform a search focused on one of the paths until it encounters a leaf node. After the leaf nodes are traced, backtracking is carried out and traced again on other paths. The last search strategy is BFS. In the BFS search strategy, the node visited first is the node with the most optimal partial solution. In the case of this herbal formula, of course, the most optimal solution is to get the most significant profit. The difference in search of the three search strategies is shown in Figure 2. The value in the circle is the profit of each node, whereas the value outside the node is the order of the node search.

### Branching strategy

**FIGURE 3:**
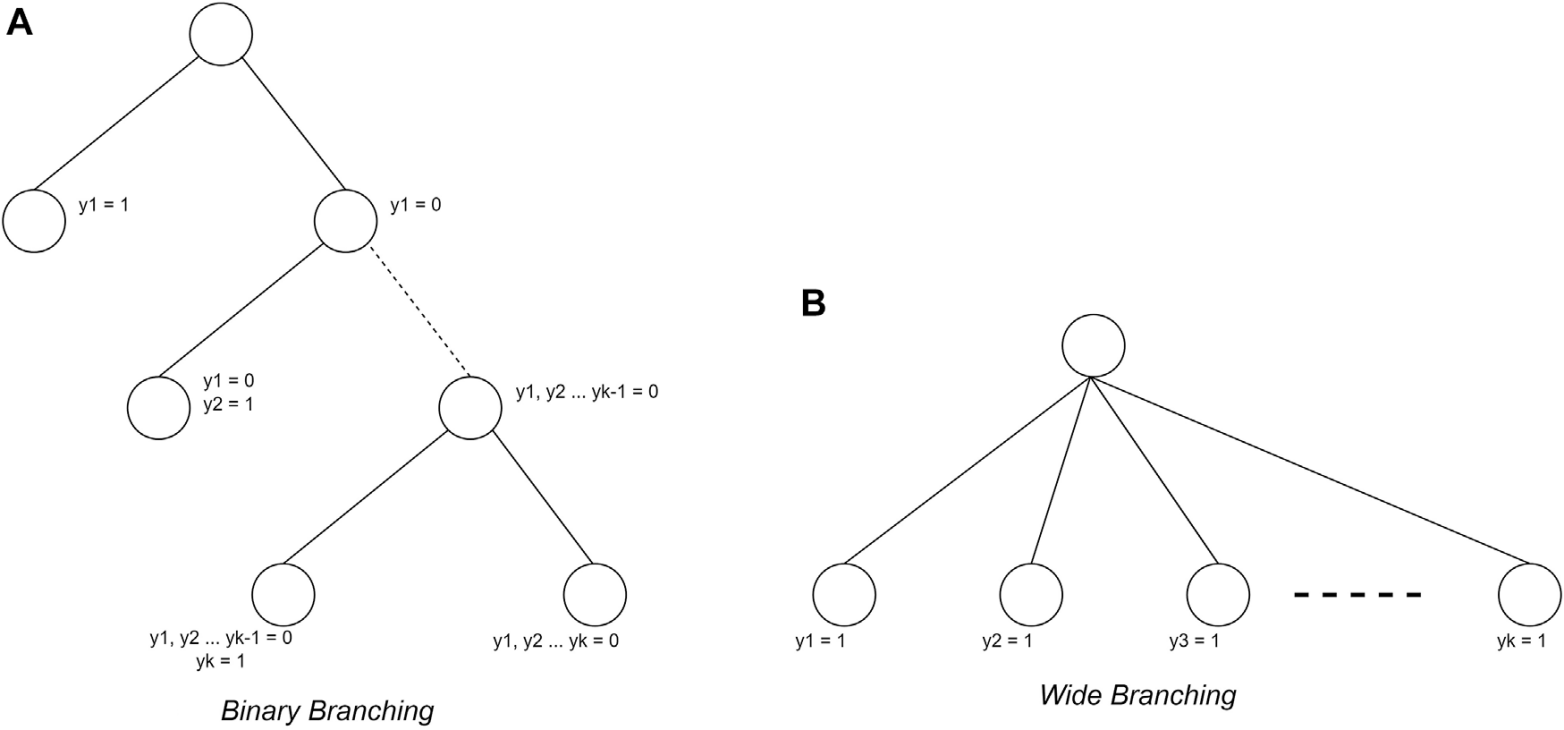
Branching strategy (Morrison et al., 2016)

In addition to determining the search strategy, the branching strategy also considerably influences its use. In this study, the branching strategy used is binary branching (Devroye, 1998; Morrison et al., 2016)) and wide branching (Morrison et al., 2016). The binary branching makes each node form two children, namely, the selected plant condition and the unselected plant condition. In the wide branching strategy, nodes will form as many children as N-level nodes as many plants are added. The different forms of the two branching strategies is shown in Figure 3.

### Pruning rule

The final aspect of the branch and bound algorithm is the pruning rule. In this study, the pruning rule used is the lower bounds to ensure the result is the most optimal solution. The lower bounds pruning rule starts by sorting the data from the most significant profit. Each node will calculate the maximum profit that may be obtained. The node will not be traversed if the profit is not greater than the maximum profit of the temporary solution.

**FIGURE 4:**
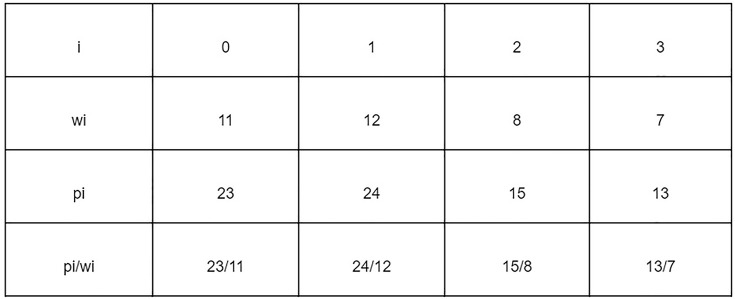
Data with weight and profit.

The problem of finding Jamu formulas can be approached as a 1–0 KNApSAcK problem, so it can be solved using the branch and bound technique (Ezugwu et al., 2019). Suppose that there are data that have weight (w_i_) and profit (p_i_) stored in an array. Furthermore, the data are sorted by the highest p_i_/w_i_ value to find the maximum profit that can be obtained with the maximum limit (W) allowed. Figure 4 shows the data that have weight and profit.

**FIGURE 5:**
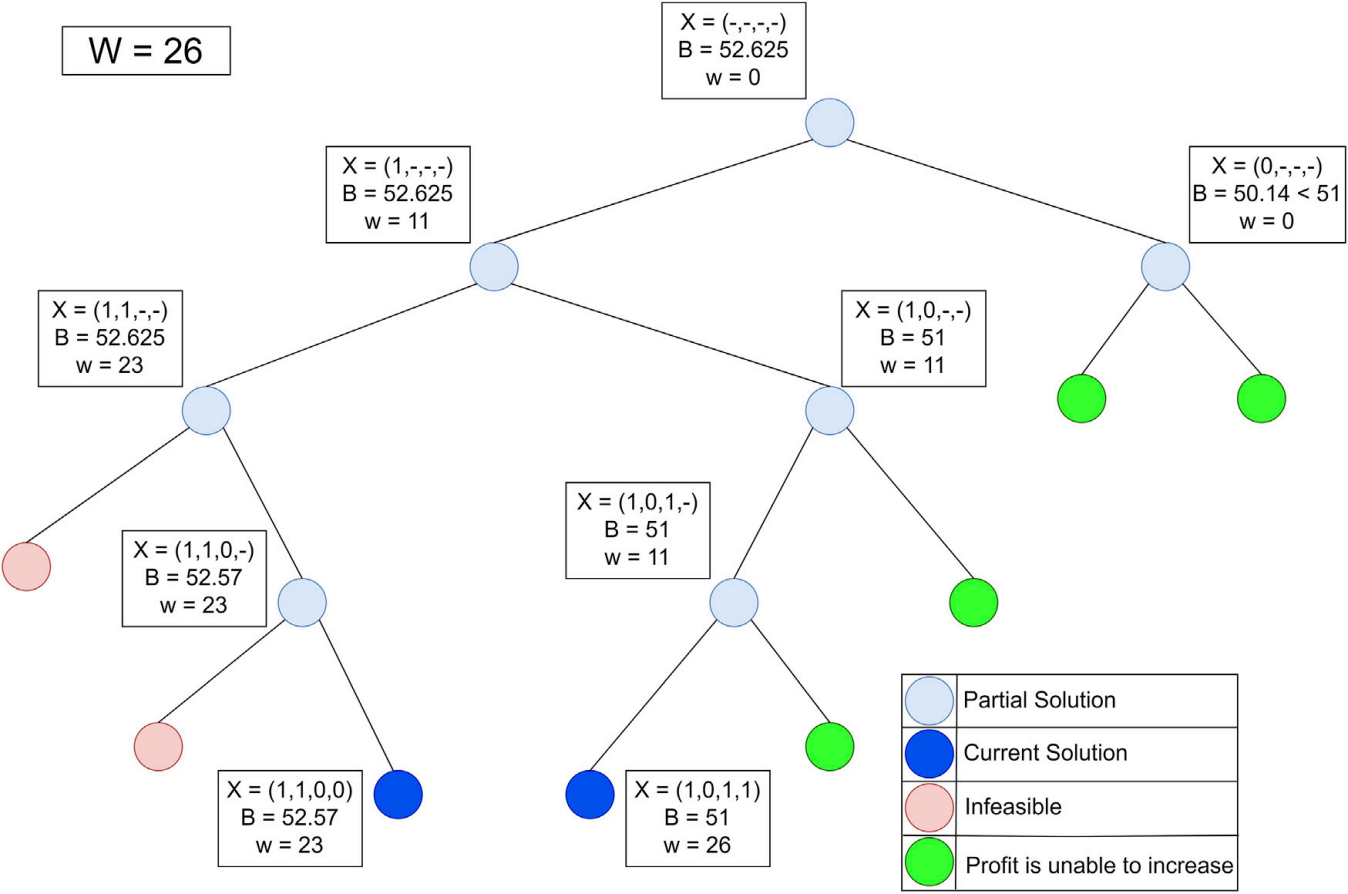
Lower bounds pruning rule.

Figure 5 shows the process of finding the maximum profit from Figure 4 using the lower bounds pruning rule. The letter X in each node represents an item that was added (1), not added (−), or not added (0) at that node. The letter B on each node is the maximum profit value obtained if the node is traced. Each node has its weight (w) based on the weight of the added items.

### Implementation of branch and bound technique

The first step is to store plant data using a struct (a collection of variable definitions wrapped in a specific name). The plant data are represented as a bipartite plant–protein graph. Each significant protein in the data has a weight taken from two centrality values, namely, BC and CC. Edges that connect plants and proteins have different weights that are taken when tracing graphs.

Examples of data stored using a struct:

′*Leucaena glauca*′: ({′3041727′: [′Ppara′, 0.4643250105, 0.9]},

{′60391226′: [′Akt1′, 0.7993787198, 0.9]},

{′13432234′: [′Pparg′, 0.6426582082, 0.9]}).

The set of plant data is stored in an array of structs. The plant struct consists of the name of the plant (name), the weight of the edge of the 14th plant protein (value), plant weight (weight), and total plant profit (totalValue) calculated from Eq. 2. After being saved, the data are sorted by the totalValue parameter. Storage using arrays allows accessing plant struct data based on array index.

#### Implementation of the branch and bound using breadth first search

**FIGURE 6:**
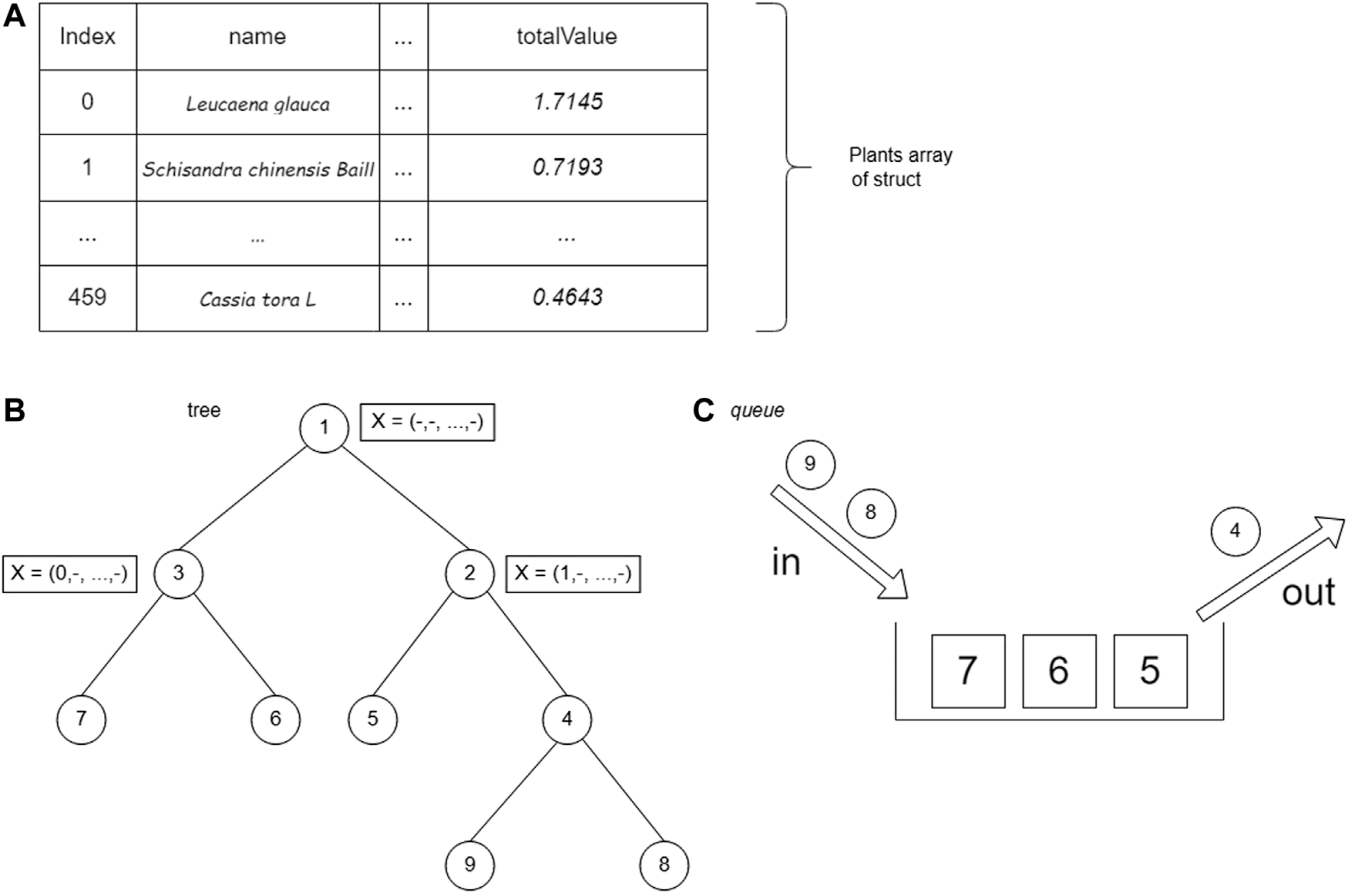
Use of the queue data structure in tree tracing.

The BrFS strategy was first tried using the queue data structure. The queue data structure is used because it has FIFO (first in, first out) properties, which follow the BrFS strategy. The BrFS strategy is implemented using the binary branching strategy and the lower bounds pruning rule. Figure 6 illustrates the use of the queue data structure in tree tracing. The node value in Figure 6 is the order of browsing in the tree. When the third node has been accessed and left the queue, the two children of that node will enter the queue. Each node in the tree will store a list of plant indexes and the totalValue of the sum of each stored plant. The X symbol in Figure 6 shows the plants stored in that node.

The branch and bound algorithm starts by generating the root node. The root node is then stored in the queue data structure. After generating the root node, the next step is to enter a loop that will stop when there are no nodes in the queue. Based on the nature of the FIFO queue data structure, data from the queue is fetched (FRONT) and removed from the queue (POP).

The next step generates the child of the node. There are two children, namely, the condition of a plant being added and that not being added. At this stage, there is a bound function call, which is a function that calculates the upper bound of a node shown in Supplementary Figure S6. After getting the bound value of the child node, the value is compared with the temporary maxProfit. If the bound value of the child node is greater than maxProfit, then the child will be stored in the queue. In addition, if the profit on the node is greater than maxProfit, then the maxProfit value will be replaced with the node’s profit.

#### Implementation of the branch and bound using depth-first search

**FIGURE 7:**
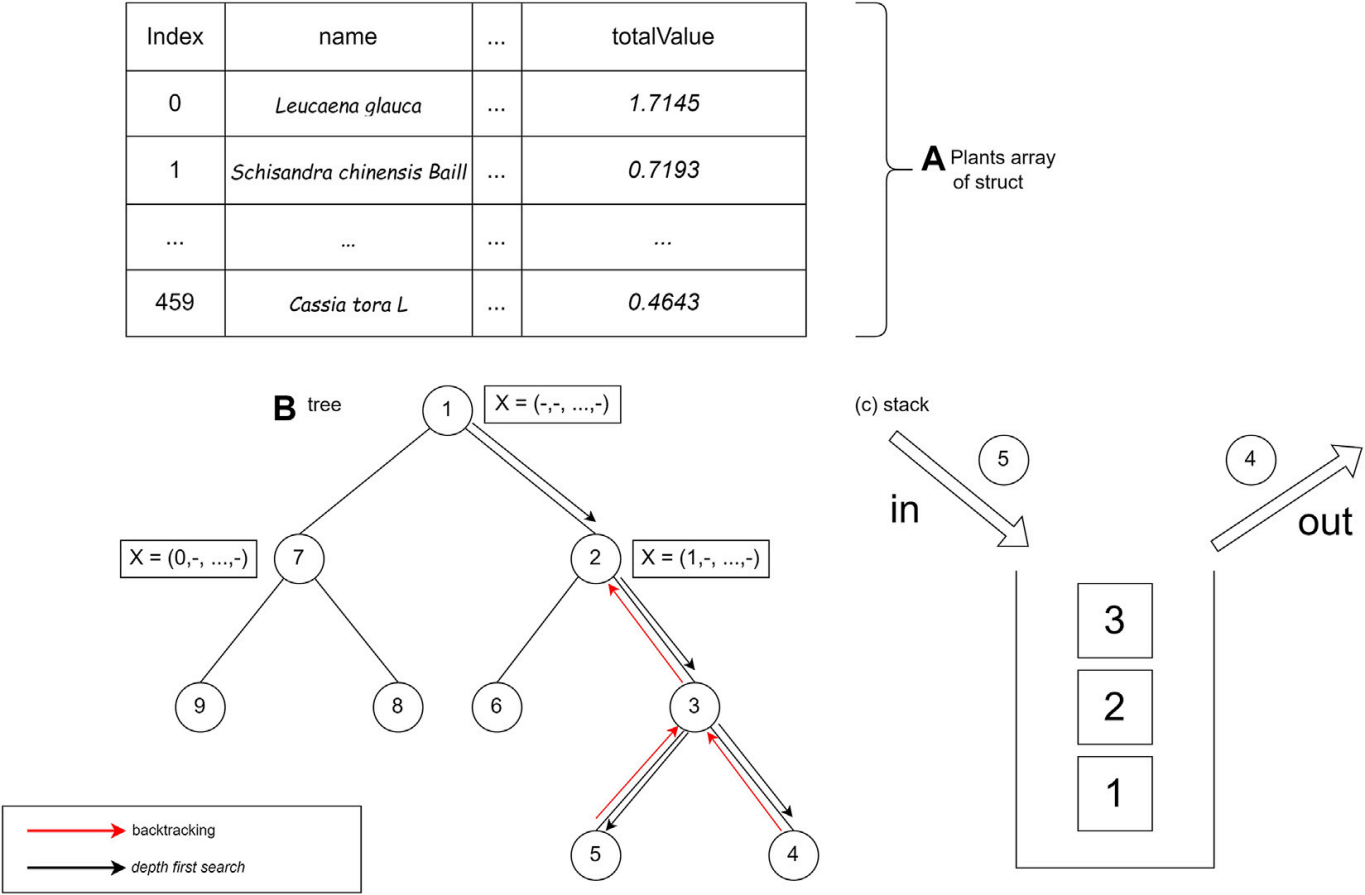
Use of the stack data structure on the tree.

The next step is implementing a DFS lookup strategy using a stack data structure. The stack data structure is used because it has first in, last out properties, which are in accordance with the DFS search strategy. The DFS strategy is implemented using a binary branching strategy and lower bounds pruning rules. The stack data structure is used in tree tracing. The value of the node is the order of tracing in the tree. The search is carried out by always prioritizing accessing the right child until it reaches the leaf node. After reaching the leaf node, backtracking is performed and traces the left child if the right child has been traced. Figure 7 shows the use of the stack data structure on the tree.

#### Implementation of the branch and bound using best-first search

**FIGURE 8:**
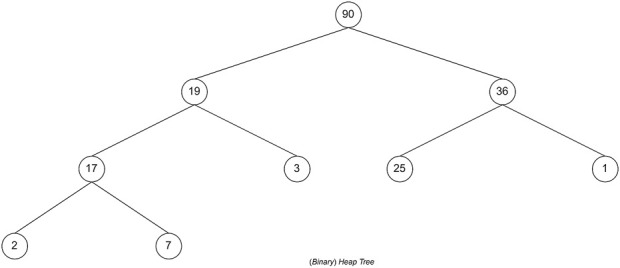
Priority queue with (binary) heap tree.

The last search strategy is BFS by using the priority queue data structure. The priority queue data structure is a data structure in the form of a (binary) heap tree. (Binary) heap tree is a data structure in the form of a complete binary tree and has the characteristic that the value of each left child and right child of a node will not be greater than its parent. The structure of the (binary) heap tree can be seen in Figure 8, where the value for each node is the total value of the nodes in the tree. The complexity of deleting data and adding data to the priority queue is O (log n), for n is the amount of data that has been stored in the priority queue, whereas the process of accessing leading data has a complexity of O (1). A priority queue follows the BFS search strategy, which will execute nodes based on the most optimal solution.

#### Using a wide branching strategy

**FIGURE 9:**
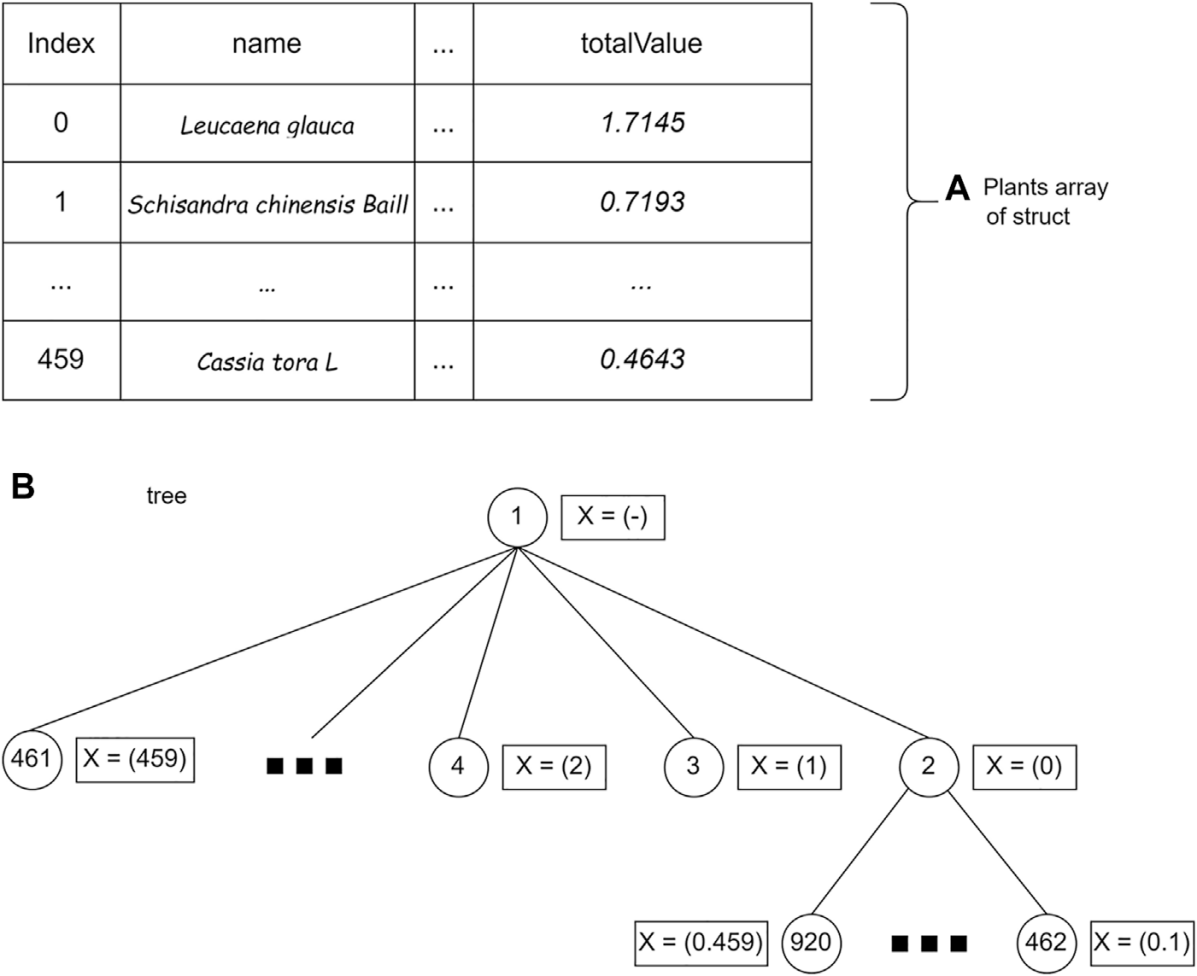
Wide branching strategy.

After implementing the binary branching strategy, a wide search strategy was implemented. In the wide branching search strategy, the BrFS strategy is used because it has the shortest computation time compared with the other two search strategies in the previous experiment. In contrast to binary branching, where each internal node must make two children for the condition of the plant being added or not, in the wide branching strategy, each node will create a different number of children depending on the plant index added last to that node. Figure 9 shows a wide branching strategy. The computation time of the wide branching strategy using BrFS is much longer than the binary branching strategy; therefore, wide branching experiments with DFS and BFS search strategies were not carried out. The X symbol in Figure 9 shows the selected crop index at each node.

## Results and discussion

### Comparison of computing time and search space

Differences in the use of search strategies and branching strategies will affect the computational time and search space. In order to get the best strategy, each computation time and search space of the strategy will be compared. Each strategy, search and branching, will get the same input data, namely, the plant–protein bipartite graph. The comparison of the search space will be seen from the number of candidate solutions generated in each strategy.

Three search strategies combined with two branching strategies resulted in four different branch and bound strategies: BrFS with binary branching, DFS with binary branching, BFS with binary branching, and BrFS with wide branching. The four strategies in the branch and bound algorithm have different computational times. Table 3 shows the complete data on the computational time for each strategy in this study: The experiment was conducted using PC with Intel Core i3 1.8 GHz processor, 6 GB RAM, SSD Sandisk 120 GB, and Linux Ubuntu 16.04 Operating System.

**TABLE 3:** Complete data on the computational time for each strategy.

The number of plant (k)	BrFS	DFS	BFS	Wide branching	Complete search
2	0.25	0.256	0.31	1.14	0.98
3	11.40	11.64	18.70	48.25	169.12
4	476.21	483.75	1070.58	2106.60	20285.02

**FIGURE 10:**
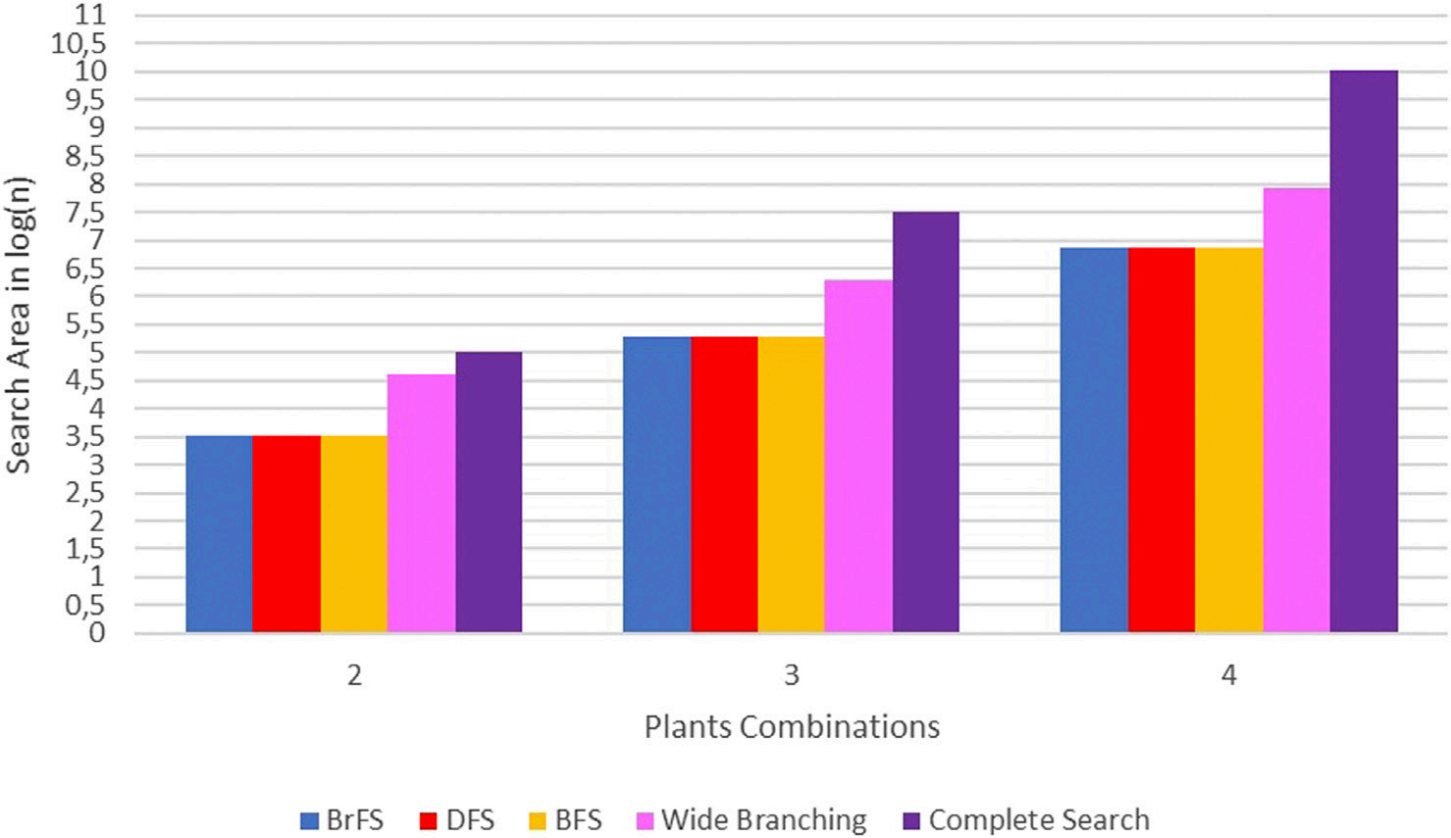
Comparison of search space area in log(n) units.

The binary search strategy produced optimum computational time, especially in the BrFS and DFS search strategies. In the combination of two plants, the longest time is the wide branching strategy, but for the combination of three and four plants, the use of complete search, as done in previous studies, requires a very long computational time. In addition to the computation time, the search space size can also be seen by calculating the number of solutions generated for each strategy. Figure 10 shows the difference in the search space size using the calculation of the number of solutions generated for each strategy in log(n) units.

In Figure 10, it can be seen that the search space of complete search is far above other strategies. The wide branching strategy has a broad search over the binary branching strategy. The binary branching strategy with either BrFS, DFS, or BFS search strategies has almost the same search area.

### Pruning process

The branch and bound and complete search algorithms have the same worst-case complexity O(T^k^), i.e., when no nodes are pruned. In this study, the branch and bound algorithm has a better computational time than the complete search strategy. It proves that the pruning process was successfully carried out in this study.

### Comparison of branching strategies

The search space and computational time of the wide branching and binary branching strategies differ quite a lot. The wide branching strategy has a longer computation time and a larger search space than the binary branching strategy because of its inability of the pruning process. Each node in the wide branching forms a very large number of child nodes and takes time and space for each level of the tree to be formed. At node level 1 will raise to 460 child nodes and will be more and more for the next node. The number of nodes at each level results in the length of the process to generate nodes with w_i_ = W, where the greater the W requested, the higher the target level.

### Search strategy comparison

In the search strategy, BFS is not better than BrFS or DFS. BFS performs a search based on the most optimum node on the node to be searched. Searching based on the most optimum node is expected to be able to cut the search space better, although it has a greater complexity when the process of deleting and adding nodes to the list. After testing, the search space of BFS with BrFS and DFS is not much different. It caused the computation time of BFS to be longer than BrFS and DFS. The best search strategy in this study is BrFS, which has a slightly better computational time difference than DFS. Searching using the BrFS strategy can reduce search space better than other strategies. The BrFS strategy of tracing nodes with the difference that the children on that node are plants are added and not added. The BrFS strategy traces nodes from the root, which nodes access high-scoring plants so that when high-scoring nodes are not added, it speeds up the process of pruning those nodes.

### Composition of k plants

From the previous search results, 460 plants had at least one target protein in T2DM. Of the 460 plants, up to four combinations will be used to create a candidate for herbal formula. For every k combination of plants, 10 candidate herbal formulas with the highest score will be taken. The higher the formula score, the more traceable T2DM target protein and the more remarkable the edge weights traced that protein.

If *k* = 1, *Mangifera indica* got the highest score with 9 out of 21 (42.8%) traceable T2DM proteins, or if seen from the formula score, 4.39 out of a maximum score of 11.3 (38.8%). The summary of the 10 candidates’ Jamu formulas for the combination of one plant with the highest score can be seen in Supplementary Table S1. Moreover, *Punica granatum* could only target seven T2DM proteins, but the formula score was higher than *Argemone mexicana*, *Salvia miltiorrhiza*, and *Daucus carota*. It shows that the edge or protein weight targeted by *Punica granatum* is greater than the three plants. A comparison of target proteins and edge weights between *Punica granatum* and *Argemone mexicana* can be seen in Supplementary Table S2.

When we compare edge weights, *Punica granatum* is relatively always higher than *Argemone mexicana*. It makes the Punica granatum formula score higher than *Argemone mexicana*, although Punica granatum cannot target *GCGR* protein. If *k* = 2, the composition of *Mangifera indica* and *Citrus aurantium* obtained the highest score, 5.26 (46.5%), and 11 T2DM proteins (52.3%) could be traced. The summary of the 10 candidate herbal formulas for the combination of two plants with the highest scores can be seen in Supplementary Table S3.

Referring to the composition of one plant, *Mangifera indica* got the highest score and could target 9 T2DM proteins. From Supplementary Table S3, it can be seen that *Mangifera indica* mostly appears in every candidate’s Jamu formula. However, the number of T2DM proteins is only approximately 10 or 11. It indicates that the second plant paired with *Mangifera indica* only added approximately two new target proteins. However, it is also possible that the edge weight of the second plant is higher than the edge weight of *Mangifera indica.* The candidates with the highest scores are *Mangifera indica* and *Citrus aurantium*. The comparison of the protein weights of the two can be seen in Supplementary Table S4. The contribution of *Citrus aurantium* is in the *KCNJ11* protein, in which the edge weight value of *Citrus aurantium* is greater than that of *Mangifera indica*. In addition, two T2DM proteins cannot be targeted by *Mangifera indica*, namely, *MTNR1B* and *EP300* proteins.

If *k* = 3, the composition of *Angelica sinensis*, *Citrus aurantium*, and *Mangifera indica* had the highest score, 5.7763 (51.1%), and there were 12 T2DM proteins (57.1%) that could be traced. A summary of the 10 best Jamu formula candidates can be seen in Supplementary Table S5. Supplementary Table S5 shows several Jamu formulas that have the same score. The plant compositions target the same T2DM protein and have the same edge weights. For a composition of three plants, the Jamu formula scores are approximately 50% of the maximum score, and all of them targeted 12 T2DM proteins.

If *k* = 4, the herbal formula candidates with the highest score are *Angelica sinensis*, *Citrus aurantium*, *Glycyrrhiza uralensis*, and *Mangifera indica*, with a score of 6.13 (54.2%), and there are 13 T2DM proteins (61.9%) that can be targeted. The 10 best candidates can be seen in Supplementary Table S6. The highest score for the composition of the three plants was the combination of Angelica sinensis, Citrus aurantium, and Mangifera indica, which can target 12 T2DM proteins. For *k* = 4, the composition reappeared as a candidate for herbal medicine with the highest score, plus the plant Glycyrrhiza uralensis. It shows that one new protein can be targeted by Glycyrrhiza uralensis but cannot be targeted by the other three plants. Glycyrrhiza uralensis consistently appeared in all 10 candidate herbs, meaning that of the four plant combinations, only Glycyrrhiza uralensis targeted a T2DM protein that neither did the other three. If traced back from the T2DM protein to the information stored in each plant, it was seen that the TCF7l2 protein was only targeted by Glycyrrhiza uralensis.

The limitation of this study is that it can only be used up to a composition of four plants. Doing a combination of five plants without reducing the number of plants will take much time. When the combination is one plant, it only takes 0.005 s for the program to finish. The combination of the two plants takes 0.34 s. Combinations of three and four plants take 253 s and 33,214 s, respectively. The comparison of the increase in program execution time is comprehensively shown in Table 4. This problem can be overcome by using parallel computing, which is beyond the scope of this study.

**TABLE 4:** Execution time for composition k plants.

Combination/Composition *k*	Time to k (sec)	Time to- (k+1)/(k) (sec)
1	0.005	68
2	0.34	744.11
3	253	131.28
4	33214	—

### Best composition of Jamu formula

The experiment results showed that the plant combinations were obtained from two plant combinations to four plant combinations. The best of each composition of plants can be seen in Table 5. The composition of the two plants consists of *Citrus aurantium* and *Mangifera indica* with a total formula score of 5.26512. In formulas for three and four plants, *Citrus aurantium* and *Mangifera indica* plants also existed. It can be concluded that *Citrus aurantium* and *Mangifera indica* plants dominate the Jamu formulas for two, three, and four plants.

**TABLE 5:** Best results of the composition of plants for the Jamu formula.

Composition of plant	Latin name	Formula score
2	*Citrus aurantium, Mangifera indica*	5.26512
3	*Angelica sinensis, Citrus aurantium, Mangifera indica*	5.77630
4	*Angelica sinensis, Citrus aurantium, Glycyrrhiza uralensis, Mangifera indica*	6.13136

The best results from the four plant compositions are *Angelica sinensis*, *Citrus aurantium*, *Glycyrrhiza uralensis*, and *Mangifera indica*. From the literature study, all the mentioned plants had the potential to be used as T2DM treatments. Li and Chen (2007) and Li et al. (2007) research analyzed the effects of Angelica sinensis polysaccharides on diabetic rats. The results showed that polysaccharides contained in *Angelica sinensis* could not only significantly reduce blood glucose levels but also improve the clinical symptoms of T2DM in the rats. Jia et al. (2015), which conducted research on the effects of Citrus aurantium in diabetic mice, reported that neohesperidin derived from Citrus aurantium helped increase oral glucose tolerance and insulin sensitivity as well as decrease insulin resistance in the diabetic mice. Moreover, aromatherapy produced from Citrus aurantium extracts also helped to relieve anxiety and fatigue in T2DM patients (Abdollahi and Mobadery 2020). Glycyrrhiza uralensis can also be used for T2DM treatment and prevention because of its flavonoids containing *α*-glycosidase and PTP1B inhibitory activities. Both inhibitions have been suggested as potential therapeutic targets for drug discovery for T2DM patients (Guo et al., 2015). As for Mangifera indica, Ngo et al. (2019) showed that its leaves extract contained potential hypoglycemic and antioxidant properties, which could be beneficial for T2DM patients, by inhibiting a starch digestive enzyme, possessing glucose uptake capacity and adsorption, and suppressing the production of nitric oxide, which its high level could cause diabetes complications. Further research is needed to verify and determine the potential efficacy of Jamu composition using *Angelica sinensis*, *Citrus aurantium*, *Glycyrrhiza uralensis*, and *Mangifera indica* plants.

## Conclusion

Bipartite graph search optimization with branch and bound algorithms for predicting Jamu formulas can reduce computation time. The complete search strategy has the worst-case and best-case complexities of O(T^k^), where T is the number of plant data, and k is the number of plant combinations. The branch and bound algorithm has the worst-case complexity of O(T^k^) and the best-case of O(T). Although the worst case is the same, the branch and bound algorithm achieves faster computation time. In this study, we found that the best branching strategy is the binary strategy t and the best search strategy are BrFS and DFS.

The proposed method suggests that the potential plant composition for the type II diabetes mellitus Jamu formula comprises *Angelica sinensis*, *Citrus aurantium*, *Glycyrrhiza uralensis*, and *Mangifera indica.* We note that this composition requires experimental validation, which is beyond our current scope. In addition, *Citrus aurantium* and *Mangifera indica* plants dominate the three- and four-plant composition for Jamu formulas. This approach is expected to be an alternative way to discover the Jamu formula more accurately.

## Data Availability

Publicly available datasets were analyzed in this study. These data can be found here: knapsackfamily.com; https://pubchem.ncbi.nlm.nih.gov/; https://pubchem.ncbi.nlm.nih.gov/bioassay/; https://chemminetools.ucr.edu/; https://www.uniprot.org/.
