# Supplementary figures and images for: Bipartite graph search optimization for type II diabetes mellitus Jamu formulation using branch and bound algorithm
Source: Front Pharmacol. 2022 Aug 11;13:978741. doi: 10.3389/fphar.2022.978741 (PMC9403330; doi:10.3389/fphar.2022.978741)

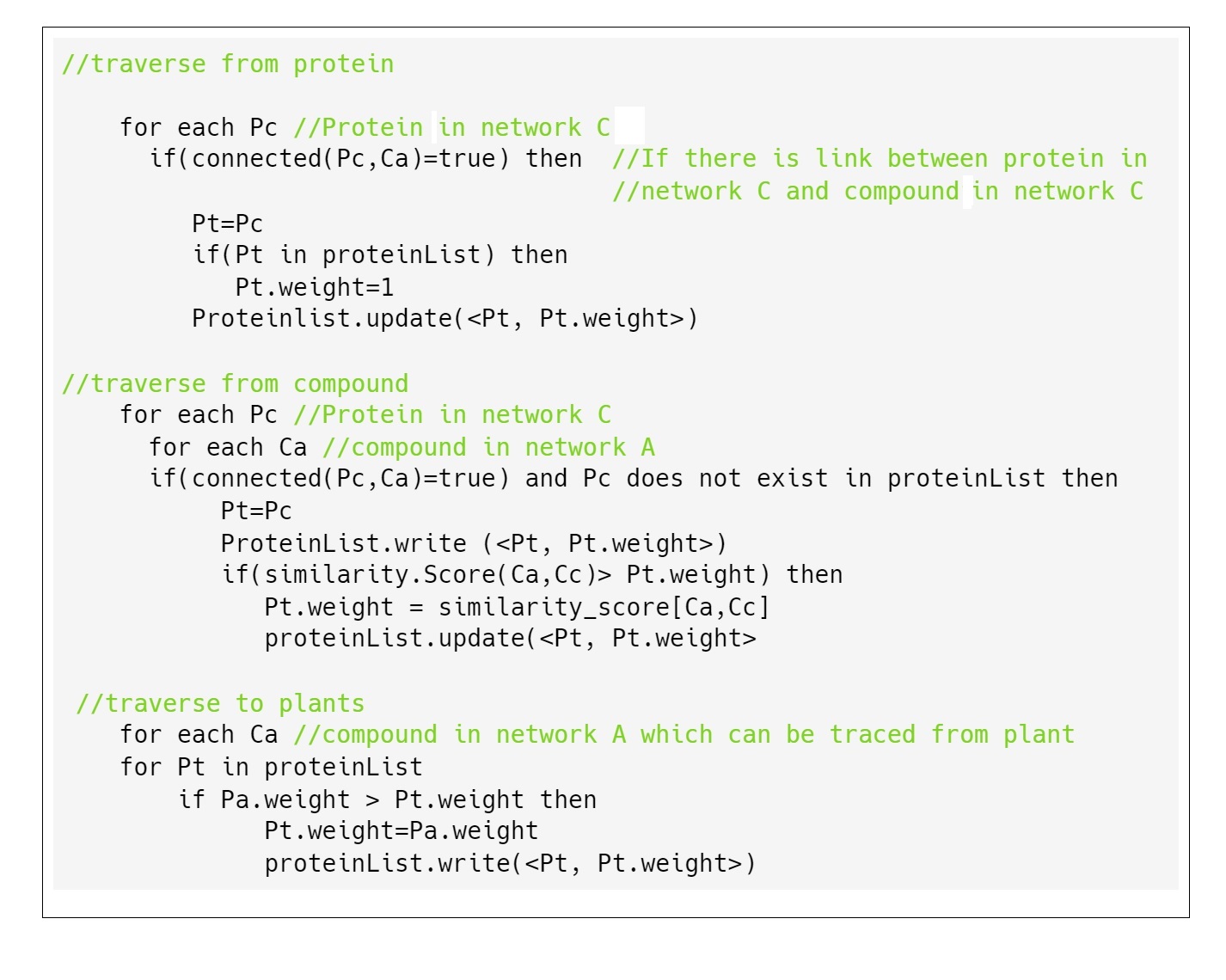

Supplement: Supplementary file 1 [file Image5.jpg]

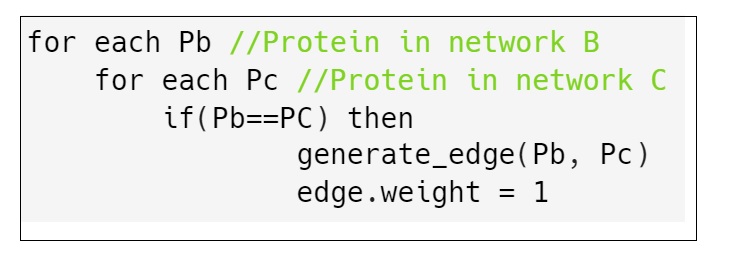

Supplement: Supplementary file 2 [file Image3.JPEG]

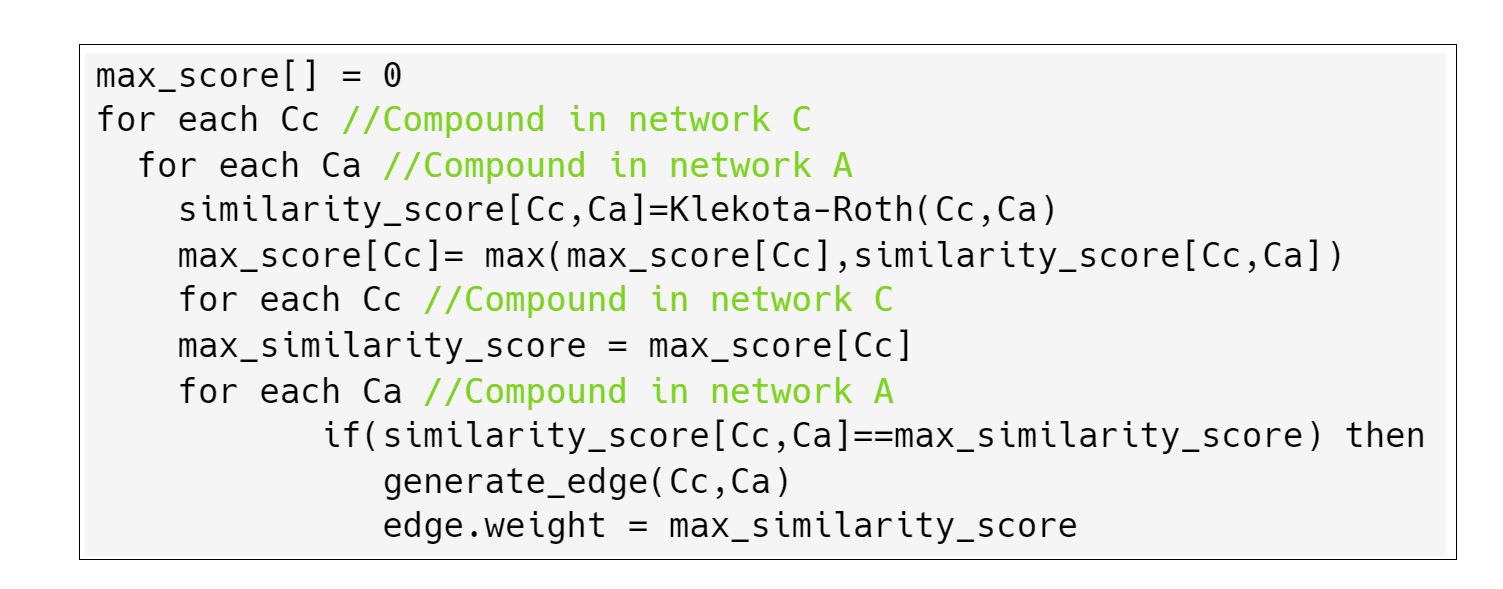

Supplement: Supplementary file 3 [file Image1.JPEG]

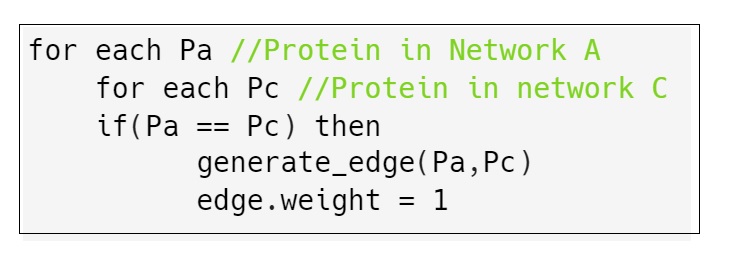

Supplement: Supplementary file 4 [file Image2.JPEG]

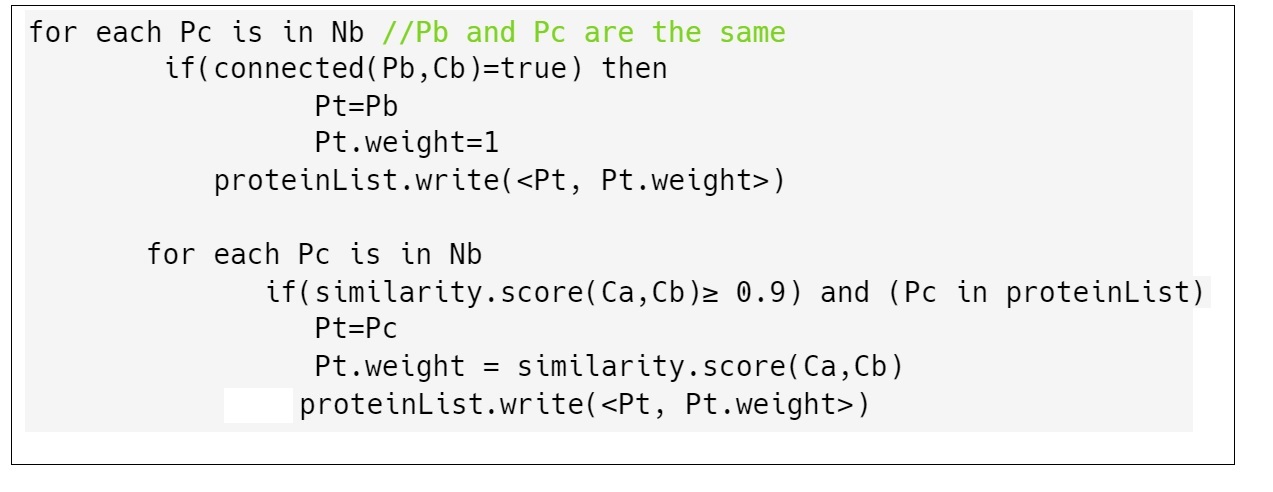

Supplement: Supplementary file 5 [file Image4.jpg]
