# Supplementary material for: Bipartite graph search optimization for type II diabetes mellitus Jamu formulation using branch and bound algorithm
Source: Front Pharmacol. 2022 Aug 11;13:978741. doi: 10.3389/fphar.2022.978741 (PMC9403330; doi:10.3389/fphar.2022.978741)
Supplement: Supplementary file 6 [file DataSheet1.docx]

Supplementary Material

# Supplementary Tables

Supplementary Table 1. Ten candidates’ Jamu Formulas for a composition of one plant with the highest formula score.

| Latin name | Formula score | Formula score in (%) | Number of target T2DM protein | Percentage of targeted T2DM protein (%) |
| --- | --- | --- | --- | --- |
| *Mangifera indica* | 4.39472 | 38.869 | 9 | 42.857 |
| *Phaseolus vulgaris* | 3.96826 | 35.097 | 9 | 42.857 |
| *Catharanthus roseus* | 3.84531 | 34.010 | 9 | 42.857 |
| *Syzygium cumini* | 3.60054 | 31.845 | 8 | 38.095 |
| *Punica granatum* | 3.59379 | 31.785 | 7 | 33.333 |
| *Argemone mexicana* | 3.54536 | 31.357 | 8 | 38.095 |

| *Salvia miltiorrhiza* | 3.52600 | 31.186 | 8 | 38.095 |
| --- | --- | --- | --- | --- |
| *Daucus carota* | 3.51318 | 31.072 | 8 | 38.095 |
| *Glycyrrhiza uralensis* | 3.41163 | 30.174 | 7 | 33.333 |
| *Zea mays L.* | 3.31603 | 29.329 | 7 | 33.333 |

Supplementary Table 2. Comparison of *Punica granatum’s* target proteins with Argemone mexicana

| T2DM Protein | Protein weight | Edge weight | |
| --- | --- | --- | --- |
|  |  | *Argemone mexicana* | *Punica granatum* |
| *PRKACA* | 0.358291 | 1 | 0.9 |
| *GCGR* | 0.585706 | 0.538462 | 0 |
| *MTNR1B* | 0.414494 | 0.542169 | 1 |
| *PPARA* | 0.464325 | 0.9 | 1 |
| *PPARG* | 0.642658 | 0.9 | 1 |
| *STAT3* | 0.377816 | 0.6375 | 0.9 |
| *AKT1* | 0.799379 | 1 | 1 |
| *INSR* | 0.61044 | 1 | 1 |

Supplementary Table 3. Ten candidates’ Jamu formulas for a composition of 2 plants with the highest formula score.

| Latin name | Formula score | Formula score in (%) | Number of target T2DM protein | | Percentage of targeted T2DM protein (%) |
| --- | --- | --- | --- | --- | --- |
| *Citrus aurantium, Mangifera indica* | 5.26512 | 46.567 | 11 | 52.381 | |
| *Angelica sinensis, Mangifera indica* | 4.97017 | 43.959 | 10 | 47.619 | |
| *Ginkgo biloba, Mangifera indica* | 4.94171 | 43.707 | 10 | 47.619 | |
| *Anacardium occidentale, Mangifera indica* | 4.93665 | 43.662 | 10 | 47.619 | |
| *Mangifera indica, Phaseolus vulgaris* | 4.92913 | 43.596 | 11 | 52.381 | |
| *Artemisia annua, Mangifera indica* | 4.89207 | 43.268 | 10 | 47.619 | |
| *Citrus sinensis, Mangifera indica* | 4.89207 | 43.268 | 10 | 47.619 | |
| *Lonicera japonica, Mangifera indica* | 4.89207 | 43.268 | 10 | 47.619 | |
| *Mangifera indica, Schizonepeta tenuifolia* | 4.89207 | 43.268 | 10 | 47.619 | |
| *Argemone mexicana, Catharanthus roseus* | 4.88972 | 43.247 | 11 | 52.381 | |

Supplementary Table 4. Comparison of edge weights of Mangifera indica and Citrus aurantium.

| T2DM Protein | Protein weight | Edge weight | |
| --- | --- | --- | --- |
|  |  | *Mangifera indica* | *Citrus aurantium* |
| *PRKACA* | 0.358291 | 0.9 | 0.9 |
| *GCGR* | 0.585706 | 0.9 | 0 |
| *KCNJ11* | 0.670335 | 0.47561 | 0 |
| *PPARA* | 0.464325 | 1 | 1 |
| *PPARG* | 0.642658 | 0.9 | 1 |
| *STAT3* | 0.377816 | 0.9 | 0 |
| *AKT1* | 0.799379 | 1 | 1 |
| *INSR* | 0.61044 | 1 | 0 |
| *INS2* | 1 | 0.433735 | 0 |
| *MTNR1B* | 0.414494 | 0 | 0.9 |
| *EP300* | 0.487221 | 0 | 0.888889 |

Supplementary Table 5. Ten candidate Jamu formulas for a composition of 3 plants with the highest score

| Latin name | Formula score | Formula score in (%) | Number of target T2DM protein | Percentage of targeted T2DM protein (%) |
| --- | --- | --- | --- | --- |
| *Angelica sinensis, Citrus aurantium, Mangifera indica* | 5.7763 | 51.088 | 12 | 57.143 |
| *Citrus aurantium, Daucus carota, Mangifera indica* | 5.67875 | 50.226 | 12 | 57.143 |
| *Citrus aurantium, Mangifera indica, Phoenix dactylifera* | 5.66678 | 50.120 | 12 | 57.143 |
| *Citrus aurantium, Mangifera indica, Polygonum cuspidatum* | 5.66678 | 50.120 | 12 | 57.143 |
| *Citrus aurantium, Mangifera indica, Polygonum multiflorum* | 5.66678 | 50.120 | 12 | 57.143 |
| *Citrus aurantium, Mangifera indica, Vitis vinifera* | 5.66678 | 50.120 | 12 | 57.143 |
| *Acorus calamus, Citrus aurantium, Mangifera indica* | 5.65461 | 50.012 | 12 | 57.143 |
| *Canarium commune, Citrus aurantium, Mangifera indica* | 5.65461 | 50.012 | 12 | 57.143 |
| *Citrus aurantium, Mangifera indica, Myristica fragrans* | 5.65461 | 50.012 | 12 | 57.143 |
| *Citrus aurantium, Mangifera indica, Piper cubeba* | 5.62661 | 49.765 | 12 | 57.143 |

Supplementary Table 6. Ten candidate formulas for Jamu composition of 4 plants with the highest scores

| Latin name | Formula score | Formula score in (%) | Number of target T2DM protein | Percentage of targeted T2DM protein (%) |
| --- | --- | --- | --- | --- |
| *Angelica sinensis, Citrus aurantium, Glycyrrhiza uralensis, Mangifera indica* | 6.13136 | 54.229 | 13 | 61.905 |
| *Citrus aurantium, Daucus carota, Glycyrrhiza uralensis, Mangifera indica* | 6.03381 | 53.366 | 13 | 61.905 |
| *Citrus aurantium, Glycyrrhiza uralensis, Mangifera indica, Phoenix dactylifera* | 6.02184 | 53.260 | 13 | 61.905 |
| *Citrus aurantium, Glycyrrhiza uralensis, Mangifera indica, Polygonum cuspidatum* | 6.02184 | 53.260 | 13 | 61.905 |
| *Citrus aurantium, Glycyrrhiza uralensis, Mangifera indica, Polygonum multiflorum* | 6.02184 | 53.260 | 13 | 61.905 |
| *Citrus aurantium, Glycyrrhiza uralensis, Mangifera indica, Vitis vinifera* | 6.02184 | 53.260 | 13 | 61.905 |
| *Acorus calamus, Citrus aurantium, Glycyrrhiza uralensis, Mangifera indica* | 6.00967 | 53.152 | 13 | 61.905 |
| *Canarium commune, Citrus aurantium, Glycyrrhiza uralensis, Mangifera indica* | 6.00967 | 53.152 | 13 | 61.905 |
| *Citrus aurantium, Glycyrrhiza uralensis, Mangifera indica, Myristica fragrans* | 6.00967 | 53.152 | 13 | 61.905 |
| *Citrus aurantium, Glycyrrhiza uralensis, Mangifera indica, Piper cubeba* | 5.98167 | 52.905 | 13 | 61.905 |
